# Characterization and tissue-specific expression patterns of the *Plasmodium chabaudi cir *multigene family

**DOI:** 10.1186/1475-2875-10-272

**Published:** 2011-09-19

**Authors:** Petra Ebbinghaus, Jürgen Krücken

**Affiliations:** 1 Institute for Parasitology and Tropical Veterinary Medicine, Freie Universität Berlin, Königsweg 67, 14163 Berlin, Germany

## Abstract

**Background:**

Variant antigens expressed on the surface of parasitized red blood cells (pRBCs) are important virulence factors of malaria parasites. Whereas *Plasmodium falciparum *erythrocyte membrane proteins 1 (PfEMP1) are responsible for sequestration of mature parasites, little is known about putative ligands mediating cytoadherence to host receptors in other *Plasmodium *species. Candidates include members of the *pir *superfamily found in the human parasite *Plasmodium vivax *(*vir*), in the simian pathogen *Plasmodium knowlesi *(*kir*) and in the rodent malarias *Plasmodium yoelii *(*yir*), *Plasmodium berghei *(*bir*) and *Plasmodium chabaudi *(*cir*). The aim of this study was to reveal a potential involvement of *cir *genes in *P. chabaudi *sequestration.

**Methods:**

Subfamilies of *cir *genes were identified by bioinformatic analyses of annotated sequence data in the *Plasmodium *Genome Database. In order to examine tissue-specific differences in the expression of cir mRNAs, RT-PCR with subfamily-specific primers was used. In total, 432 cDNA clones derived from six different tissues were sequenced to characterize the transcribed *cir *gene repertoire. To confirm differences in transcription profiles of *cir *genes, restriction fragment length polymorphism (RFLP) analyses were performed to compare different host tissues and to identify changes during the course of *P. chabaudi *infections in immunocompetent mice.

**Results:**

The phylogenetic analysis of annotated *P. chabaudi *putative CIR proteins identified two major subfamilies. Comparison of transcribed *cir *genes from six different tissues revealed significant differences in the frequency clones belonging to individual *cir *gene subgroups were obtained from different tissues. Further hints of difference in the transcription of *cir *genes in individual tissues were obtained by RFLP. Whereas only minimal changes in the transcription pattern of *cir *genes could be detected during the developmental cycle of the parasites, switching to expression of other *cir *genes during the course of an infection was observed around or after peak parasitemia.

**Conclusions:**

The tissue-specific expression of cir mRNAs found in this study indicates correlation between expression of CIR antigens and distribution of parasites in inner organs. Together with comparable results for other members of the *pir *superfamily this suggests a role of *cir *and other *pir *genes in antigenic variation and sequestration of malaria parasites.

## Background

Antigenic variation is a major characteristic of malaria parasites of the genus *Plasmodium *leading to severe and chronic infections in a variety of vertebrates. Malaria parasites have developed strategies to evade host immune responses by expressing a large and diverse repertoire of variant proteins on the surface of parasitized red blood cells (pRBCs) [1-4]. By rapidly switching between these antigens, the parasites avoid antibody-mediated immunity of the host thus enabling the parasites to proliferate in the host without being completely eliminated by the adaptive immune response. Furthermore, these surface antigens were proposed to be involved in adherence to endothelial cells causing sequestration of late trophozoites and schizonts in post-capillary venules in specific inner organs, which considerably contributes to severe pathology in the host [5-8].

In the most virulent human pathogen *Plasmodium falciparum*, the PfEMP1 protein family encoded by widely studied *var *genes was shown to be expressed at the surface of pRBC mediating the parasite binding to endothelial cells lining small blood vessels [9,10]. Several *in vitro *assays with *P. falciparum *revealed a tight regulation of the expression of the individual variant antigens by silencing, activation and mutually exclusive expression, thus only one variant protein is expressed in parasites at any given time [10-13]. By switching the expression to another variant, antigenic properties of the surface of pRBCs changes and the parasites prevent their complete clearance [10,14]. Typical waves of parasitaemia in persistent *P. falciparum *infections reflect the repeated switching between different members of the diverse repertoire of variant antigens [10].

Further variant surface antigens encoded by multigene families were found in several other *Plasmodium *species infecting humans, monkeys and rodents [4,15-17]. However, homologues of the *var *genes are not present in *Plasmodium vivax *or other malaria species [18], with exception of several *var*-like sequences from the chimpanzee parasite *Plasmodium reichenowi *[19], a close relative of *P. falciparum*.

The largest multigene family in *Plasmodium *genomes was described to be formed by the *Plasmodium interspersed repeat *(*pir*) genes [20,21]. The superfamily of *pir *genes constitutes the major variant surface antigen family in most *Plasmodium *species. They were found in the human pathogen *P. vivax *(*vir*) [22], in the simian parasite *Plasmodium knowlesi *(*kir*) [18] and in the rodent malaria species *Plasmodium yoelii *(*yir*), *Plasmodium berghei *(*bir*) and *Plasmodium chabaudi *(*cir*) [15,17,23]. Transcriptional changes of *yir *genes modulated by host immunity were reported in immunocompetent mice infected with *P. yoelii *[24]. In contrast to the exclusive expression of individual *var *genes and consecutive activation of different genes in *P. falciparum*, transcriptional profiling analyses in *P. yoelii *showed a simultaneous expression of a broad range of different *yir *genes within different intra-erythrocytic developmental parasite stages [25]. In a single parasite, however, only one to three *yir *transcripts were detectable. Cunningham and colleagues [25] concluded that antigenic variation in *P. yoelii *probably involves exposure of the immune system to many different YIR antigens and transcriptional switching takes place without any epigenetic memory. For the variant VIR proteins of the most widely distributed human malaria pathogen *P. vivax*, a similar differential expression in natural infections could also be detected [26] and for the *cir *genes of the rodent malaria species *P. chabaudi *antigenic switching in laboratory mice has already been described [15].

Although the PIR proteins were localised close to the surface of pRBCs infected with *P. vivax, P. yoelii, P. berghei*, and *P. chabaudi *[20,22,24,27], less is known about the role of PIR proteins in host/parasite interactions. For instance, a supposed correlation of antigenic variation of the PIRs with sequestration in inner organs has not yet been analysed. In *P. falciparum*, adherence of pRBCs to different host receptor such as CD36, ICAM-1 or chondroitin-sulfate A (CSA) is mediated by the major variant protein PfEMP1 [6,8,28], but no homologues to these proteins were detected in most other malaria pathogens. Therefore, it is conceivable that the PIR proteins, which are also expressed on the surface membrane of pRBCs, are as well involved in adherence to endothelial cells and sequestration of pRBCs in the microvasculature of inner organs.

The complex phenomenon of sequestration is hitherto not completely understood as indicated by the common assumption that sequestration of mature pRBCs in the microvasculature of the host tissues, as found in *P. falciparum *infections, does not occur for *P. vivax*. Thus, it has been assumed that the human parasite *P. vivax *must have developed different strategies e.g. adherence to barrier cells in the spleen to avoid spleen clearance [29]. Recently, however, *in vitro *assays provided new evidence for a cytoadherence of *P. vivax *pRBCs to endothelial cells and placental cryosections suggesting a cytoadherence comparable to that of *P. falciparum *pRBCs *in vivo *[30]. Direct or indirect evidences for an involvement of VIR antigens in binding to host receptors are currently still missing. In the most studied rodent model *P. berghei*, real-time *in vivo *imaging of transgenic parasites revealed CD36-mediated sequestration of schizonts in adipose tissues and lung as well as an accumulation of schizonts in the spleen [31]. Accumulation of other blood stages was also observed in different tissues including the brain and placenta of pregnant mice [32-34]. Nevertheless, identification of parasite ligands involved in binding to CD36 or other host receptors are still missing. *In vitro *adherence of *P. chabaudi *infected erythrocytes to purified human CD36 has been observed [35]. Moreover, sequestration to microvascular endothelial cells was reported to appear in an organ specific manner in *P. chabaudi *infections, predominantly in liver, but also in brain and spleen thus resembling at least partially the sequestration pattern in *P. falciparum *infections.

In this study, the relationship between antigenic variation of the *cir *multigene family and accumulation of parasites localization in different host tissues was analysed at the molecular level. Bioinformatic characterization of the *cir *genes was used to identify different subfamilies within the multigene family. First indications for a tissue-specific expression of *cir *genes were obtained by amplification of a representative sample of transcribed *cir *genes from different host tissues. Transcriptional profiling analyses of *cir *genes in different host tissues using RFLP analysis of RT-PCR products provided further evidence of a tissue-specific expression of *cir *genes in *P. chabaudi *infections. These results provide first indications for a possible correlation of antigenic variation of the *cir *multigene family and sequestration in host tissues in *P. chabaudi *infections.

## Methods

### Bioinformatics

All annotated sequence data of putative *cir *genes in the *Plasmodium *Genome Database Resource version 8.0 [36] (PlasmoDB; plasmodb.org) were compiled (Additional file 1). For confirmation of correct annotation, a CD-BLAST [37-39] was performed to identify and locate the conserved domain of the CIR-BIR-YIR superfamily [22,23] (Pfam protein families database accession number [PF06022]). Sequences with a partial CIR-BIR-YIR conserved domain were excluded from all subsequent analyses.

For phylogenetic reconstruction, putative CIR proteins were aligned using ClustalW2 [40] and a phylogenetic tree was calculated with PhyML 3.0 [41] using maximum likelihood estimation and the JTT model [42] for amino acid substitution. The gamma shape parameter and the proportion of invariable sites were estimated and the number of substitution rate categories was set to four. The implemented BIONJ algorithm was used to build the starting tree. Resulting trees (Newick format) were visualised and processed with MEGA4 [43,44].

For validation of structural protein motifs such as transmembrane domains, signal peptides and PEXEL motifs, analyses of the protein sequences were examined with Protscale [45], TMHMM 2.0 [46,47] and SignalP [48]. Results were compared with annotations extracted from PlasmoDB.

### Mice

All experiments were performed with 5-8 weeks-old outbred female NMRI mice (Crl:NMRI(Han)) provided by Charles River (Sulzfeld, Germany). The mice were kept in cages with a maximum of five animals per cage and received food and water *ad libitum*. The experiments were planed according to all relevant guidelines for animal protection and approved by German authorities responsible for animal protection.

### Infection of mice

A non-clonal line of *P. chabaudi *very similar but not identical to *P. chabaudi *AS was used [49-51]. Blood stages of *P. chabaudi *were weekly passaged in female NMRI mice by intraperitoneal injection (i.p.) of a droplet of tail vein blood diluted in PBS. Parasitaemia was evaluated in Giemsa-stained blood smears and total erythrocytes number was counted in a Neubauer chamber.

For the experiments, blood of an infected NMRI mouse was collected by cardiac puncture under anesthesia. For each transcriptional profiling experiment, six mice were infected i.p. with approximately 100 parasitized red blood cells (pRBCs). Organs and blood were collected at about 30% parasitaemia, i.e. just before peak parasitaemia.

For transcriptional profiling during the course of an infection, tail vein blood of mice infected i.p. with 100 pRBCs was passaged i.p. into naïve female NMRI mice at days 7 (early infection), 14 (around peak parasitaemia), 21 (7 days after peak parasitaemia) and 35 (21 days after peak parasitaemia). Expression of *cir *genes was analysed when parasitaemia reached about 30%.

### RNA and DNA extraction

Blood samples of *P. chabaudi *infected mice were collected by cardiac puncture, rapidly frozen and stored at -80°C. Small pieces of liver, spleen, kidneys, lung and brain were transferred into RNA Later (Sigma Aldrich) and kept at -80°C for long-term storage. Total RNA was extracted using NucleoSpin^® ^RNA II kit (Macherey-Nagel) according to the manufactures instructions. Genomic DNA extraction was performed with the NucleoSpin^® ^Blood kit (Macherey-Nagel).

### Verification of complete *cir *gene structures

To verify the complete gene structure of selected *cir *genes, RT-PCR and genomic PCR were performed in parallel. The primer pairs used for amplification and detailed information about PCR conditions can be found in Additional file 2.

### Cloning of RT-PCR products and sequencing

Residual contaminating genomic DNA in total RNA preparations was digested with DNase I (Fermentas). First strand cDNA was synthesised using 1 μg RNA and the RevertAid™ Premium Reverse Transcriptase (Fermentas) with Oligo dT_18 _primers including reactions without reverse transcriptase as negative controls for amplification.

For transcription analysis, specific primers spanning the second exon encoding an essential part of the CIR-BIR-YIR domain (> 98% of its length) were designed for both major *cir *gene subfamilies. The primer pairs f1up (5'-AATACGCTATTTTATGGTTTAGTTATAAA-3')/f1lo (5'-TGAAATTCCTAAAATAATGGGTATTATTAAAA-3') and f2up (5'-TATGCTATTTTATGGTTAAGTTATATGCTA-3')/f2lo (5'-ATGAATACTTATAAGCAATTCCCAAGAAAA-3') were targeted to highly conserved sequence regions for amplification of a broad range of *cir *genes.

Following cDNA synthesis, amplification with the AccuPrime™ DNA polymerase (Invitrogen) was performed using subfamily-specific primers for *cir *subfamily 1 and 2. After an initial denaturation for 30 s at 94°C, 40 cycles of 10 s at 94°C, 30 s at 55°C, 30 s at 72°C followed by a final extension of 10 min at 72°C. RT-PCR products were then isolated from a 0.8% agarose gel and precipitated in the presence of glycogen.

In order to sample a first repertoire of transcribed *P. chabaudi cir *genes, RT-PCR products of each tissue (blood, liver, spleen, kidney, lung and brain) were gel-purified and cloned into the pCR™4-TOPO^® ^TA vector (Invitrogen). Thirty-six clones for each tissue and subfamily were sequenced (GATC, Constance, Germany) resulting in sequences for 216 independent cDNA clones for *cir *subfamily 1 and 2.

### Transcriptional profiling of RT-PCR products by RFLP

For transcriptional profiling, DNase digestion of RNA and cDNA synthesis was performed as described before and RT-PCR products were amplified with the Phusion^® ^Hot Start II High-Fidelity DNA Polymerase (Fermentas) using subfamily-specific primers for *cir *subfamily 1 and 2. After an initial denaturation for 30 s at 98°C, 50 cycles of 10 s at 98°C, 30 s at 55°C, 30 s at 72°C were performed followed by a final extension for 10 min at 72°C.

Gel-purified RT-PCR products (150 ng) were digested with *Alu*I or *Xap*I (Fermentas) in 5 μl for 3 h at 37°C followed by enzyme inactivation for 20 min at 65°C. The restricted fragments (30 ng) were analysed with the Agilent 2100 Bioanalyzer using the DNA 1000 LabChip^® ^kit (Agilent) following the manufactures instructions. In order to ensure reproducibility of the RFLP profiles, both RT-PCR and RFPL were usually repeated at least twice resulting in 4 replicates.

### Statistical analyses

In order to evaluate whether clones representing certain *cir *subfamilies were significantly more frequently recovered from one tissue than from others, frequencies were compared using a Z-test.

## Results

### Phylogenetic analyses of putative CIR proteins in PlasmoDB

**Figure 1:**
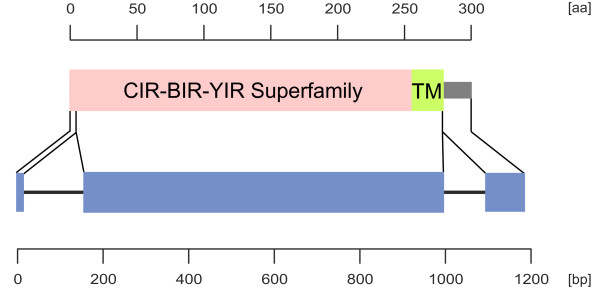
**Schematic domain architecture and genomic organization of putative CIR proteins**. For a representative CIR protein of cir subfmily 1 and 2 the protein domain architecture is shown in the upper and exon/intron structure in the lower panel. The protein and gene sequences are presented by lines, protein domains and exons are indicated by boxes. Separate scale bars are given for protein and gene structure, respectively. The CIR-BIR-YIR Superfamily (pink, accession number [PF06022] for the Pfam database) includes CIR, BIR and YIR proteins of the *Plasmodium *species *P. chabaudi, P. berghei *and *P. yoelii *exhibiting a conserved domain of a consensus sequence of 279 amino acids which is encoded by the first and second exon of the conserved three exon structure of these *pir *genes [22,23]. The COOH-terminal transmembrane domain (TM), shown as green box. is still part of the CIR-BIR-YIR domain. Exons are indicated by blue boxes.

A total of 199 putative CIR proteins had been annotated in the *Plasmodium *Genome Database Resource (state September 2011) as *cir *genes. For confirmation of correct annotation, the putative CIR sequences were examined regarding the conserved domain of the CIR-BIR-YIR Superfamily (accession number [PF06022] for the Pfam database). This conserved sequence motif were found in several CIR, BIR and YIR proteins of the *Plasmodium *species *P. chabaudi, P. berghei *and *P. yoelii *(Figure 1) [22,23]. The presence of a complete conserved domain of the CIR-BIR-YIR superfamily could only be confirmed for 186 of them. For the remaining 13 putative CIR proteins, no (four sequences) or only a partial CIR-BIR-YIR conserved domain (seven sequences) could be identified. As there was no evidence for the missing regions in the adjacent genomic sequences, these database entries most like represent *cir *pseudogene fragments. These sequences were excluded from all subsequent analyses. Furthermore, three sequences (PCHAS_040020; PCHAS_000130; PCHAS_000400) showed two hits for the CIR-BIR-YIR conserved domain. In these cases the first hit only represented a partial domain and these partial domains were therefore also excluded. The final 186 putative CIR proteins containing a complete conserved CIR-BIR-YIR domain were used for all following analyses and are listed in Additional File 1. Due to the fact that the *P. chabaudi *genome is assembled already to 14 (more or less) complete chromosomes whereas the *P*. *yoelii *genomic sequence is still scattered on 5.617 unassembled contigs, it is highly probable that vast majority of the *cir *genes in the genome have been identified and annotated in the current PlasmoDB release (8.0) and that the number of *cir *genes is indeed much smaller than the number of *yir *genes (about 838 genes) [52].

**Figure 2:**
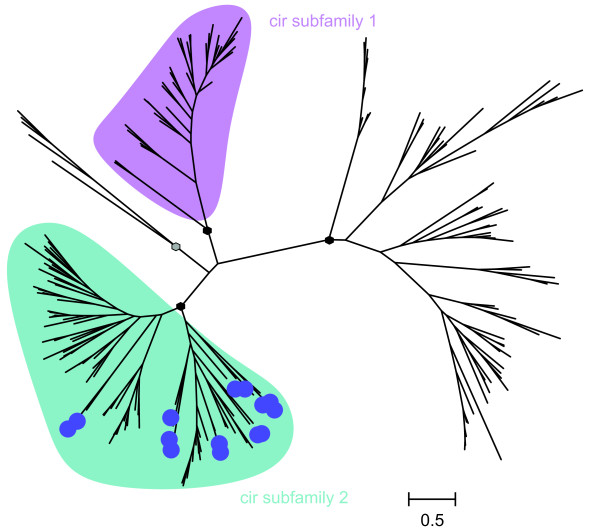
**Phylogenetic tree of 186 annotated CIR and selected YIR proteins**. Sequences of 186 CIR-BIR-YIR conserved domains of the putative CIR proteins and 14 different putative conserved domains of YIRs representing different YIR subfamily proteins were aligned using ClustalW2 [40] and a maximum likelihood tree was calculated using PhyML [41]. The cir subfamilies, indicated by the unrooted phylogenetic tree, are highlighted by different colours. Subfamily 1 (purple) and subfamily 2 (cyan) consist of CIR proteins very similar to well known YIR and BIR proteins of the PIR superfamily [20]. The remaining CIR proteins were more divergent, often due to highly variable insertions within the CIR-BIR-YIR conserved domain. These sequences were finally not assigned to any subfamily. The phylogenetic tree indicated that the YIR proteins cluster only with subfamily 2 and neither with subfamily 1 nor the large CIR proteins. The putative YIR domains are indicated by blue circles. The scale bar represents 0.5 substitutions per amino acid position. Black hexagons mark major branches with more than 95% support, the grey hexagon labels the minor branch with 92% support. See in Additional file 3 for identification of the individual proteins in each branch.

The phylogenetic maximum likelihood analysis of all 186 annotated CIR domains including a selected subset of the related YIR proteins in *P. yoelii *using representatives of all different subgroups defined previously in the *yir *family [52] (Figure 2) identified four distinct branches. Three large branches of CIR proteins were indicated with likelihood ratios of more than 95% as statistical support values at the nodes and one small branch with 92% support. Two of these branches were designated here as subfamily 1 and 2 containing CIR amino acid sequences that show a relatively low variability within the subfamilies and are very similar to well known YIR and BIR proteins in terms of size and overall structure. The other two branches with more divergent CIR proteins were not designated as a specific subfamily due to their high heterogeneity. In contrast to subfamily 1 and 2, these unassigned branches include many very long CIRs. In most cases these proteins exhibit an additional highly variable insertion within the CIR-BIR-YIR domain. Such divergent family members have already been described in previous studies of the PIRs superfamily [20]. Interestingly, the analysis also reveals important information about the relationship between CIR and YIR proteins. The phylogenetic tree indicated that the subset of YIR proteins shares similarities only with the predicted subfamily 2 but neither with subfamily 1 nor the unassigned heterogeneous CIR sequences.

For identification of the individual proteins in each branch, the phylogenetic tree of CIR and YIR proteins is given in Newick format in Additional file 3.

### Localization motifs found in CIR proteins

For protein structural analyses, the 186 putative CIR proteins were examined in regard to subcellular localization characteristics, such as putative signal peptides, transmembrane domains, and the PEXEL motif (*Plasmodium *export element) [53,54]. Results were then compared to annotations in PlasmoDB. Detailed information on protein motifs identified in all putative CIR proteins can be obtained from Additional file 1. In only one of the 186 sequences (PCHAS_114740) a signal peptide had been annotated, however, the re-analysis with SignalP revealed only a 1.6% probability for the presence of a signal peptide in this particular predicted CIR protein with a maximum cleavage site probability of 1.2% between amino acid position 26 and 27. Moreover, a cDNA sequence encoding a corresponding CIR protein with signal peptide could not be amplified by RT-PCR using primers derived from PCHAS_114740 in the non-clonal *P. chabaudi *line used throughout this study. Altogether, this indicates that the CIR proteins lack a signal peptide and presumably use alternative pathways for transport out of the parasite cell across the parasitophorous vacuole to the host cell membrane.

**Figure 3:**
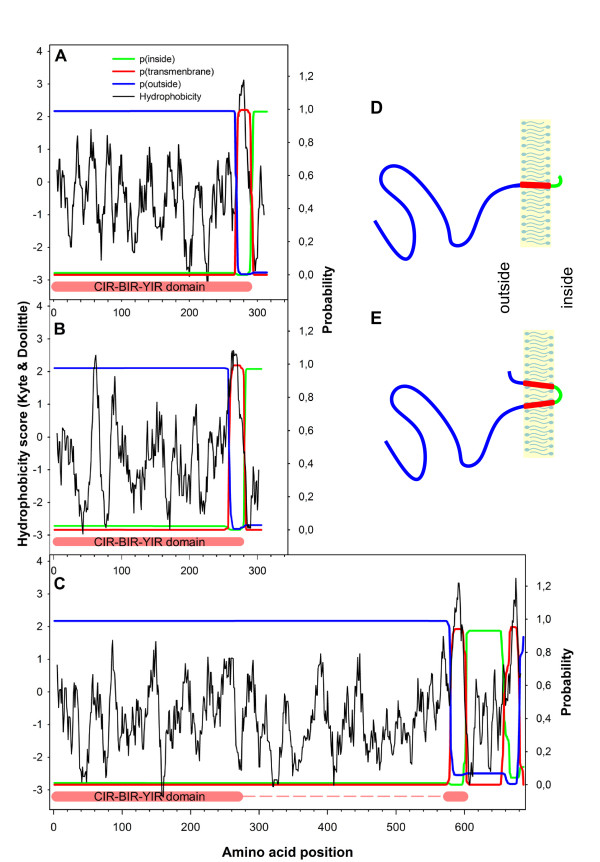
**Transmembrane topology of some selected CIR proteins**. To examine the predicted transmembrane topology of selected CIR Proteins two different analyses tools were used. With the Protscale analysis, hydrophobicity plots were created according Kyte & Doolite [45]. With the TMHMMM 2.0 server [47], probabilities of predicted transmembrane topologies were calculated. For three different CIR proteins (A-C) the results of the Protscale and TMHMM 2.0 were here compiled in one graph. On the x-axis the amino acid positions are given whereas on the left y-axis the hydrophobicity scores and on the right y-axes the probabilities for extracellular, intracellular and transmembrane localization are shown, respectively. The CIR proteins PCHAS_060060 of subfamily 1 (A) and PCHAS_000320 of subfamily 2 (B) were analysed as well as one of the unassigned CIR protein (PCHAS_010040) (C). For the first two proteins, only one transmembrane helix was predicted with more than 99% probability immediately close to the COOH-terminus of the CIR-BIR-YIR conserved domain, whereas the long NH_2_-termininal part was assumed to be located outside. For the longer unassigned CIR protein, an additional second helix was located behind the CIR-BIR-YIR conserved domain close to the COOH-terminus in this CIR. The large NH_2_-terminus as well as less than 10 amino acids of the COOH-terminus were predicted to be outside. The individual curves were highlighted by different colours (see graphic legend). Additionally, schematic presentations of the transmembrane topologies of subfamily 1 and 2 CIR proteins are schematically shown in (D) while topology of a CIR protein with two predicted transmembrane helices is given in (E).

For the vast majority of putative CIR proteins (147 sequences) the presence of one transmembrane domain had been annotated in PlasmoDB. Hydrophobicity plot analyses according to Kyte and Doolittle were performed using Protscale [45] confirming a hydrophobic stretch close to the COOH-terminus of these CIRs. A prediction of transmembrane topology using TMHMM 2.0 [47] indicated a transmembrane helix of approximately 23 amino acid residues immediately upstream of the COOH-terminus of the CIR-BIR-YIR conserved domain with a probability of more than 99%. The largest part of the NH_2_-terminus of CIR proteins was predicted to be located outside and only less than 30 amino acids inside the cytoplasm. The corresponding hydrophobicity plots as well as the predicted transmembrane topology for representative CIR proteins of subfamily 1 and 2 are shown in Figure 3A and 3B. For a notably lower proportion of CIR proteins (37 sequences) two transmembrane helices and for one CIR sequence (PCHAS_011490) even three transmembrane helices had been annotated. The phylogenetic analysis revealed that all these protein sequences were unassigned CIRs and did not belong to subfamily 1 or 2. The hydrophobicity analyses indicated an additional second hydrophobic stretch behind the CIR-BIR-YIR conserved domain immediately at the COOH-terminus in these CIRs. The predicted transmembrane topology by TMHMM also suggested the presence of two transmembrane helices, both spanning the membrane with 23 amino acids. In this case not only the large NH_2_-terminal CIR domain but also a segment of less than 10 amino acids at the COOH-terminus were predicted to be outside (Figure 3C). For the putative sequence PCHAS_011490 with three predicted transmembrane domains, two additional helices were found behind the CIR-BIR-YIR conserved domain by Protscale. The schematic transmembrane topology for selected CIR proteins is given in Figure 3D and 3E.

Finally, the putative CIR proteins were scanned for the conserved five-residue PEXEL motif Rx[L/I]x[E/Q/D] with the Protein Motif Pattern search tool in PlasmoDB. This short hydrophobic peptide has been proposed to be localised 16-24 amino acids downstream of the NH_2_-terminus and to mediate the export of proteins across the parasitophorous vacuole to the erythrocyte cytoplasm [53,54]. Seventeen sequences of the putative CIR proteins were identified to have the specific PEXEL motif but none of these sequences exhibited the PEXEL motif at its canonical position. In the protein sequence PCHAS_114700 the motif is located at amino acid position 4-8 and in four sequences between position 30-70. In another 10 small CIR proteins and in two large CIR proteins, the PEXEL motif is found between the amino acids 90-300 or 640-660, much closer to the COOH- than to the NH_2_-terminus. Hence, all annotated putative CIR proteins only possess an Rx[L/I]x[E/Q/D] stretch in their sequence but lack a functioning PEXEL motif.

### Verification of complete gene structures

**Figure 4:**
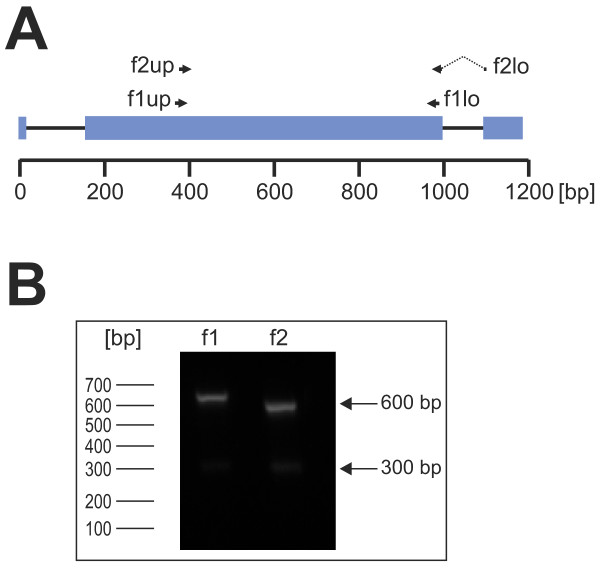
**RT-PCR with subfamily-specific primers**. (A) The *cir *gene structure and the location of the subfamily-specific primer pairs for cir subfamily 1 and 2. The f1up/f1lo (subfamily 1) and f2/f2lo (subfamily 2) primers are indicated as black arrows. Most *cir *genes consist of 3 exons (blue rectangles) with about 15 bp, 800-840 bp (highly polymorphic with highly conserved motifs), and 80-90 bp coding sequence, respectively, and two introns (100-150 bp) in conserved positions. (B) RT-PCR products of cir subfamily 1 (f1) and 2 (f2). As an example for all RT-PCR products used in the following RFLP, the RT-PCR products obtained from blood of a female NMRI mouse infected with 100 pRBCs (parasitaemia about 30%) are shown. Expected product sizes were about 600 bp. An additional smaller but much weaker RT-PCR product (approx. 300 bp) appeared in most of the amplifications which was revealed by sequencing as a further putative *cir *gene splice variant. These smaller cir products were excluded from all following RFLPs and only the expected RT-PCR products of about 600 bp were purified and digested. For size determination a 100 bp ladder was used.

In several studies, a common three-exon gene structure was postulated for genes of the *pir *superfamily in rodent malaria parasites [15,23,24]. For verification of the complete gene structure of selected *cir *genes of subfamily 1 and 2, further bioinformatic analyses and experimental tests such as parallel RT- and genomic PCR were performed. The results show that indeed the *cir *genes consists of three exons like the related *yir *genes of *P. yoelii *[25,52]. The coding region of cir cDNAs is unevenly distributed between these exons with about 15 bp of the open reading frame in the first, 800-840 bp (highly polymorphic but containing highly conserved motifs) in the second, and 80-90 bp in the third exon. The two introns of 100-150 bp are found in conserved positions (Figure 4). Moreover, evidence for the occurrence of minor splice variants of *cir *genes was detected by comparison of RT-PCR and genomic PCR products. Transcripts of *cir *genes were amplified where alternative splicing of the first exon was detected leading to an NH_2_-terminally truncated putative protein. In this case a 'cryptic' 5' splice-site within the non-coding region of the first exon was used whereas the 3' splice-site was identical in both transcripts (Additional file 4). Whether this splice variant encodes a functionally important CIR protein variant or represents only a mis-spliced product can not finally be decided from the available data. Sequences of genomic DNA and full-length cDNA sequences have been deposited in GenBank with accession numbers [GenBank:JF904729 - JF904735] (Additional file 2).

### RT-PCR of *cir *genes with subfamily-specific primers

For the subsequent transcriptional profiling analyses (see below), subfamily-specific primers for cir subfamily 1 and subfamily 2 were designed spanning the second exon, the essential part of the CIR-BIR-YIR conserved domain. The primer pairs were located at highly conserved sequence regions for amplification of a broad range of *cir *cDNAs (Figure 4A). The expected RT-PCR product sizes for subfamily 1 and subfamily 2 were about 600 bp (Figure 4B). In most PCRs, however, an additional smaller but very faint RT-PCR product (approx. 300 bp) appeared. Sequencing analyses indicated that this product is a notably smaller cir transcript with a deletion of about 250 bp within the second exon. This cDNA most likely also represents an alternatively spliced *cir *variant, in particular since this cDNA [GenBank:JF969288] has an uninterrupted open reading frame.

### Cloning of cir cDNAs from individual tissues

To examine whether the expression of certain cir antigen mRNAs correlates with the localization of the parasites in particular internal organs, a first repertoire of transcribed *cir *genes was amplified from parasites using RT-PCR and subfamily-specific primers for subfamilies 1 and 2. For this purpose, a single female NMRI mouse was i.p. infected with 100 pRBCs and, at a parasitaemia of 30%, blood, liver, spleen, kidney, lung and brain were collected for RNA isolation. RT-PCR products of about 600 bp from each tissue were excised from agarose gels and directly cloned into the pCR4-TOPO vector. For each tissue, clones were obtained from at least three independent RT-PCR reactions. Thirty-six clones for each tissue and subfamily were sequenced resulting in 216 cir sequences for each cir subfamily. Information about individual sequences with GenBank^® ^accession numbers can be found in Additional file 5. A broad range of different sequences from both subfamilies was amplified as shown by phylogenetic maximum likelihood analysis of the deduced protein sequences in comparison with the 186 putative conserved domains obtained from PlasmoDB (Additional file 6) indicating that the method is able to amplify a representative repertoire of both subfamilies. The facts that (i) the length of the amplified fragments differs only minimally (mean length and SD is 561 bp ± 2.5% for subfamily 1 and 523.7 bp ± 4.4% for subfamily 2) and (ii) the percent identity is very high (mean percent identity and SD is 80.8% ± 8.5% for subfamily 1 and 76.7% ± 10.9% for subfamily 2) a strong bias introduced by unequal amplification efficacies is at least unlikely.

**Figure 5:**
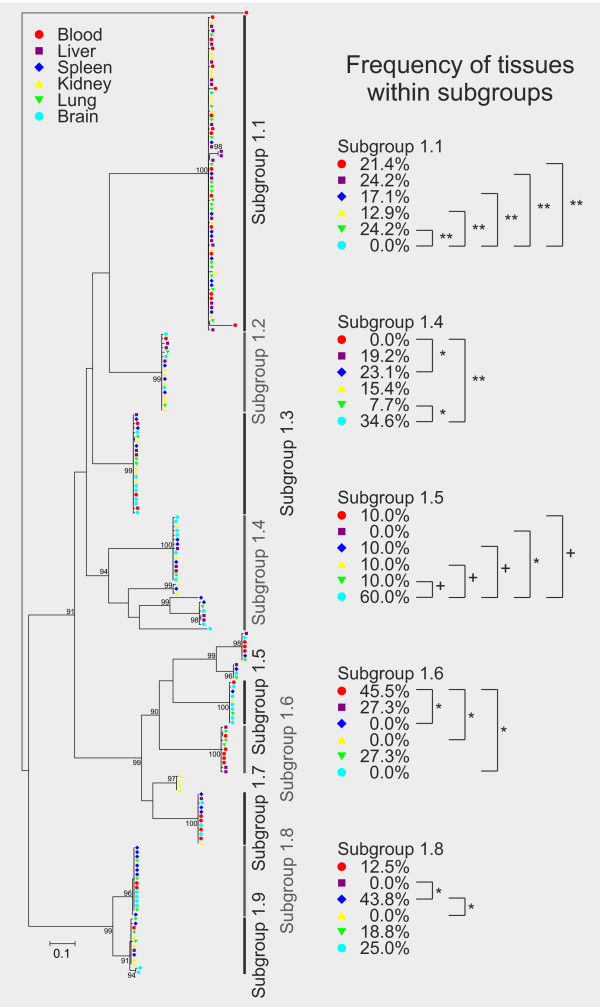
**Phylogenetic tree representing frequency of tissues within subgroups of the cir subfamily 1**. Deduced protein sequences from 216 amplified cir cDNAs of subfamily 1 amplified from six different host tissues of a single female NMRI mouse infected with 100 pRBCs (parasitaemia about 30%) were aligned using ClustalW2 [40] and a maximum likelihood tree was calculated using PhyML [41]. RT-PCR products for each tissue (blood, liver, spleen, kidney, lung and brain) were cloned and 36 clones from each tissue were sequenced. Statistical support is indicated as likelihood ratios only for those nodes with at least 90% support. The scale bar represents 0.1 substitutions per amino acid position. The origins of sequences (host tissues) are indicated by different colours as shown in the figure graphic legend. The cir subfamily 1 repertoire expressed here can be divided in 9 subgroups. Five of these subgroups (subgroup 1.1, 1.4, 1.5, 1.6, 1.8) showed significant differences in frequency of tissues. The p-values were calculated by Z-test; **p < 0.01; *p < 0.05; ^+^p < 0.07). Small (rarely occurring) subgroups of cir cDNAs were excluded from the statistical analysis. For identification of the individual sequences in each branch see Additional file 7.

The phylogenetic analyses of the proteins deduced from subfamily 1 cir cDNA sequences identified nine different sequence subgroups within the repertoire of expressed subfamily 1 *cir *genes. Branch lengths and likelihood ratios for nodes with at least 90% support indicated that the transcribed cir sequences within these subgroups are very similar or even identical (Figure 5). See in Additional file 7 for the Newick format of the phylogenetic tree of cir subfamily 1.

Statistical analyses using a Z-test were done to identify significant differences in the frequency of different tissues represented within those individual subfamilies that are sufficiently represented within the total population. The method does not allow to detect expression differences for minor cir transcripts. Results are also indicated in Figure 5. Most remarkably, the cir cDNA belonging to subgroup 1.1 could be frequently recovered from liver (24.2% of all subgroup 1.1 clones), lung, (24.2%) blood (21.4%), spleen (17.1%) and kidney (12.9%) but not from brain (0%). The absence of this cir cDNA from our brain samples is statistically significant when compared to all other tissues (p < 0.01). In contrast, the cir cDNA sequences of subgroup 1.4 were not found in blood cDNA but significantly more often present in brain (34.6%; p < 0.01) and spleen (23.1%; p < 0.05) samples. Furthermore, a significant difference could be detected between brain and lung (7.7%) in subgroup 1.4 (p < 0.05). The cir cDNAs in subgroup 1.5 were absent from liver samples (0%) but a higher number (60%) of these clones came from brain samples than from all other tissues investigated (10% for each tissue). While the differences between brain and liver were statistically significant (p < 0.05), differences between brain and the other tissues (10%) did just not reach significance (p < 0.07). The cDNA sequences of subgroup 1.6 were absent from spleen, kidney and brain samples but were found in blood (45.5%), liver (27.3%) and lung (27.3%). The frequency of clones obtained from blood within the cir subgroup 1.6 were significantly higher when compared to spleen, kidney and brain (p < 0.01). Finally, for subgroup 1.8 no cir cDNAs could be detected in liver and kidney. In contrast, this subgroup was obtained with a high frequency of 43.8% from the spleen and with intermediate frequencies of 12.5% from blood, 18.8% from lung and 25.0% from brain. However, only the differences between the frequencies for spleen, liver and kidney were statistically significant (p < 0.05).

**Figure 6:**
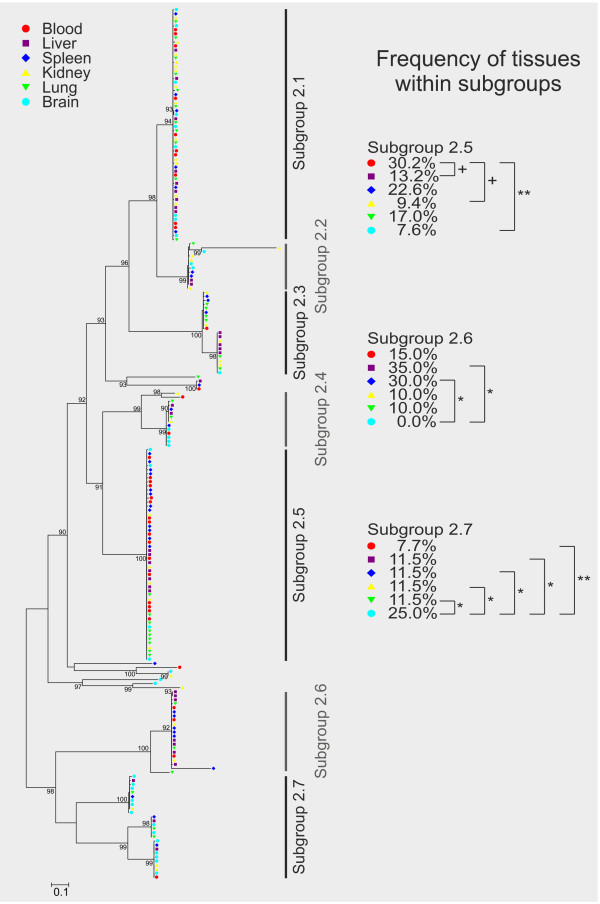
**Phylogenetic tree representing frequency of tissues within subgroups of the cir subfamily 2**. Phylogenetic tree based on maximum likelihood estimation showing 216 protein sequences deduced from cir cDNAs of subfamily 2 amplified from six different host tissues of a single female NMRI mouse infected with 100 pRBCs (parasitaemia about 30%). The RT-PCR products for each tissue (blood, liver, spleen, kidney, lung and heart) were cloned and 36 clones from each tissue were sequenced. Statistical support is indicated as likelihood ratios only for those nodes with at least 90% support. The scale bar represents 0.1 substitutions per amino acid position. The origins of sequences (host tissues) are indicated by different colours as shown in the figure graphic legend. The cir subfamily 2 repertoire expressed here can be divided in 7 subgroups but only three of these subgroups (subgroup 2.5, 2.6, 2.7) showed significant differences of the frequency between tissues. The p-values were calculated by Z-test; **p < 0.01; *p < 0.05; ^+^p < 0.07). Small (rarely occurring) subgroups of cir cDNAs were excluded from the statistical analysis. For identification of the individual sequences in each branch see Additional file 8.

An equivalent analysis was done for the *cir *subfamily 2 (Figure 6). However, only minor differences in tissue distribution of cir cDNAs could be found within the 7 subgroups identified in the expressed set of subfamily 2 *cir *genes. Significant differences in the frequency between tissues were shown only in three subgroups of cir subfamily 2. In subgroup 2.5, a significant difference was observed only between blood with a frequency of 30.2% and brain with a frequency of 7.6% (p < 0.01). Differences between blood and liver (13.2%) as well as blood and kidney (9.4%) did just not reach significance (p < 0.07). In subgroup 2.6, the absence of cDNAs obtained from brain resulted in a statistical difference only to the frequency of clones derived from liver and spleen samples with frequencies of 35.0% and 30%, respectively (p < 0.05). Within the cir cDNA sequences in subgroup 2.7, frequency of clones recovered from brain was significantly increased in brain (25%) compared to all other tissues with p < 0.01 for blood (7.7%) and p < 0.05 for liver (11.5%), spleen (11.5%), kidney (11.5%), and lung (11.5%). See in Additional file 8 for the Newick format of the phylogenetic tree of cir subfamily 2.

All these differences in tissue distribution of the transcribed cir cDNAs found in this cloning and sequencing study are first indications for a tissues specific expression of *cir *genes and were analysed in more detail in the subsequent experiments.

### Expression profiling of *cir *genes in different host tissues

Due to these first differences in the expression of *cir *genes, further analyses of different transcription profiles in various host tissues during the infection were carried out using RFLP of RT-PCR products in order to confirm differences in expression of *cir *genes between different host tissues for a larger number of mice.

**Figure 7:**
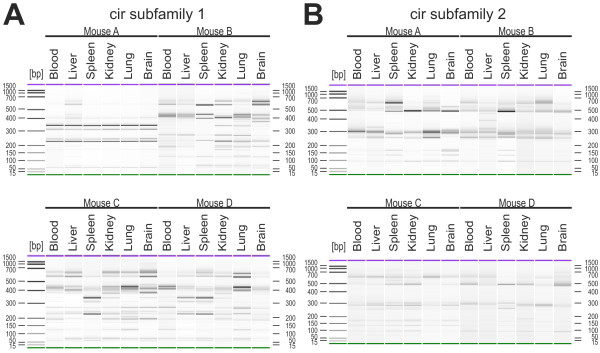
**Expression profile analyses of *cir *genes in different tissues of infected mice by RT-PCR RFLP**. For RFLP analyses, organs and blood were collected at a parasitaemia of 30% from female NMRI mice infected with 100 pRBCs. After RT-PCR amplification for the six host tissues with the subfamily-specific primers, 150 ng of purified RT-PCR products (approx. 600 bp) were digested with *Alu*I. The DNA fragments (30 ng) were analysed using the DNA 1000 kit for the Agilent 2100 bioanalyzer for accurate sizing. Compared are RT-PCR RFLP profiles of blood, liver, spleen, kidney, lung and brain for four mice for cir subfamily 1 (A) and cir subfamily 2 (B). DNA 1000 bp ladder was used and in each lane an upper (1000 bp, purple) and a lower (25 bp, green) marker are indicated.

Therefore, six mice were infected i.p. with 100 pRBCs. Only four out of these six mice developed a patent infection indicating that 100 pRBCs i.p. is close to the minimal infectious dose and that the initial diversity of parasites is kept at a minimum using this infection protocol. Organs and blood were again collected at about 30% parasitaemia, i.e. just before peak parasitaemia. After amplification using the subfamily 1 and subfamily 2 specific primers, PCR products were digested with the restriction enzyme *Alu*I and analysed using the DNA 1000 kit for the Agilent 2100 bioanalyzer for accurate and reproducible separation and size determination (Figure 7).

Comparing the RFLP profiles, considerable differences in the banding patterns of the individual organs for subfamily 1 and 2 were observed thus confirming differences in expression of *cir *mRNAs between different host tissues. Nevertheless, the prominent fragments observed in RFLP agreed in many, though not all cases well with the restriction pattern obtained *in silico *from the sequences of the cir clones described above (see Additional file 9). This similarity of experimental RFLP patterns and the *in silico *predicted restriction patterns of some cir cDNA sequences of the cloning and sequencing experiment suggested at least a similar repertoire of amplified *cir *genes in both infection experiments. On the other hand, none of the profile pattern was identical between the four mice infected with 100 *P. chabaudi *pRBCs taken from the same parental population. This indicates that random selection of 100 pRBCs as starter population from a large parental population severely influences the initial *cir *gene expression pattern. Despite the fact that patterns for organs differed within individual mice, there was no clear tissue-specific pattern detectable that was conserved between mice.

For subfamily 1, no transcriptional differences between the tissues were found in mouse A (Figure 7A). Moreover, the restriction pattern obtained for this mouse was highly different in comparison to the restriction profiles of mice B-D and the sizes of the individual fragments did not clearly refer to any *in silico *fragments of cir sequences obtained in the cloning study. This presence or absence of individual fragments in the RT-PCR RFLP profiles for subfamily 1 are further hints for a differential expression of *cir *genes in different host tissues. In particular, in mouse B, additional fragments in restriction patterns were observed e.g. in brain (~ 370 bp) or in kidney and lung (~ 340 bp) which were absent from samples of all other tissues (Figure 7A). Further differences within the transcriptional profiles of cir subfamily 1 were detected e.g. in the spleen of mouse C, showing a distinct banding pattern compared to the other five tissues of the same mouse.

Differences - though generally less pronounced - were also found in cir subfamily 2 (Figure 7B). An additional fragment of ~ 400 bp was present e.g. in the liver of mouse B which was absent in blood, spleen kidney, lung, and brain samples from the same mouse. Furthermore, two additional bands of cir fragments of approximately 100-200 bp appeared in the spleen of mouse B.

In conclusion, the differences in the transcriptional profiles of the different host tissues found in this experiment confirmed a correlation between parasite tissue localization on expression of specific *cir *genes.

### Expression profiling of *cir *genes during the course of infection

**Figure 8:**
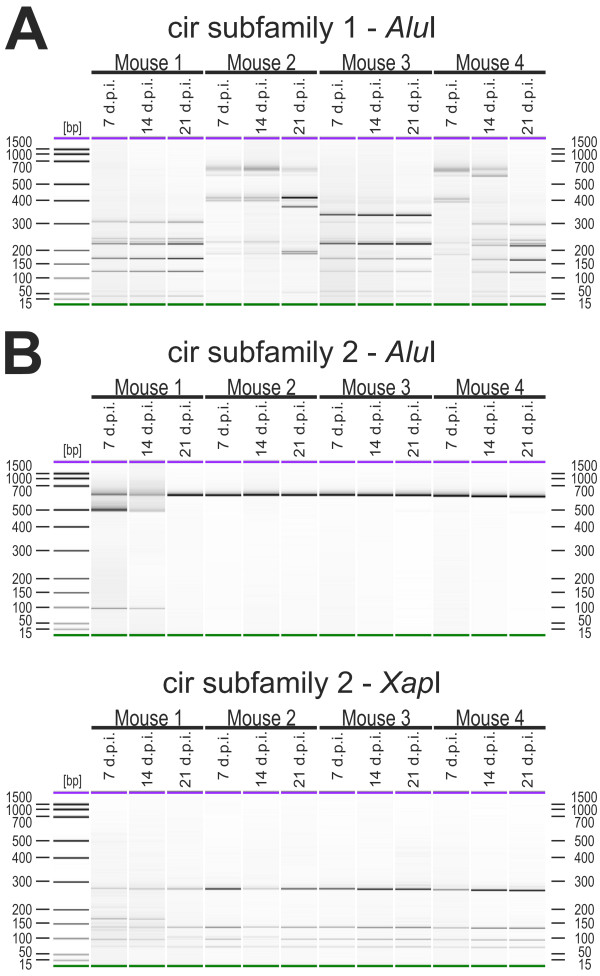
**Transcriptional changes of *cir *gene expression during the course of infection**. For RFLP analyses of expression changes of the *cir *genes during the course of infection, blood of female NMRI mice infected with 100 pRBCs were passaged on days 7, 14 and 21 days post infections (d.p.i.) into naïve female NMRI mice. Blood of these passaged mice was again collected at 30% parasitaemia. After amplification using the subfamily-specific primers for both subfamilies, 150 ng of purified RT-PCR products (approx. 600 bp) were digested with *Alu*I and 30 ng were then analysed using the DNA 1000 kit for the Agilent 2100 bioanalyzer. The RT-PCR RFLP profiles for four mice of subfamily 1 are presented in (A). The restriction digest of subfamily 2 are shown in the upper panel of (B) where only at day 7 p.i. and day 14 p.i. of mouse 1 a few smaller restriction fragments could be detected. Therefore a second digest with *Xap*I for 3 h at 37°C was performed (B, lower panel). DNA 1000 bp ladder was used and in each lane an upper (1000 bp, purple) and a lower (25 bp, green) marker are indicated.

In the blood of mice infected with 100 pRBCs, changes in the expression pattern of *cir *genes during the course of infection could also be detected by RT-PCR RFLP (Figure 8). The tail vein blood of mice infected i.p. with 100 pRBCs was passaged into naïve female NMRI mice at days 7 (early infection), 14 (around peak parasitaemia), 21 (7 days after peak parasitaemia) and 35 (21 days after peak parasitaemia) p.i. For analyses, blood of these passage mice was collected again at about 30% parasitaemia, i.e. just before peak parasitaemia.

Surprisingly, only two out of four immunocompetent mice (mouse 2 and 4) showed changes in expression of subfamily 1 *cir *genes in the blood between day 7 (early infection), day 14 (around peak parasitaemia) and day 21 (after peak parasitaemia) (Figure 8A) p.i. However, the virtually identical patterns produced here provide additional evidence for the reproducibility of the RFLP method. In mouse 2, a completely different restriction fragment pattern was observed at day 21 p.i., seven days after peak parasitaemia, compared to the profiles of early parasitaemia (day 7 p.i.) and peak parasitaemia (day 14 p.i.). In mouse 4 the first differences in the expression pattern of the *cir *genes were observed already at peak parasitaemia (day 14 p.i.) and a completely changed RFLP pattern was found at day 21 p.i. (after parasitaemia) compared to day 7 p.i. in the early phase of infection. Surprisingly, however, no changes in the expression pattern could be observed for subfamily 1 in mouse 1 and 3 (Figure 8A) and for all four mice in subfamily 2. The same RFLP pattern of amplified *cir *cDNAs could be identified throughout the course of an infection (Figure 8B). Since *Alu*I did apparently not cut the cir subfamily 2 members expressed in this experiment, an additional restriction digestion with *Xap*I was then performed (Figure 8B) confirming further that no changes in expression of cir subfamily 2 occurred in the course of an infection in these mice.

At day 35 p.i., all mice had completely resolved the infection irrespectively of changes in *cir *gene expression pattern before. Apparently, a chronic infection of the mice failed to occur after i.p. infection with 100 pRBC whether or not changes in *cir *gene expression pattern were detectable in the earlier infection.

### Transcriptional profiling of *cir *genes throughout intraerythrocytic development

For RFLP analyses of transcriptional changes of the *cir *genes at different life cycle stages, 30 μl tail vein blood of female NMRI mice infected with 100 pRBCs was collected at 3 h (early trophozoites), 10 h (late trophozoites) and 17 h (mature trophozoites and early schizonts) after beginning of the light cycle on day 13 p.i. (parasitaemia about 30%).

**Figure 9:**
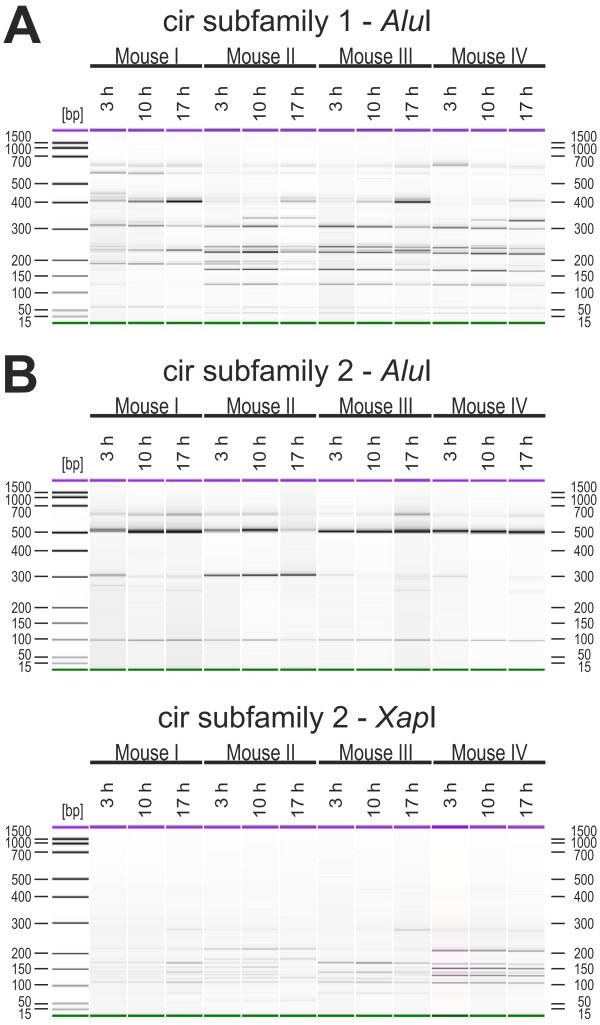
**Transcriptional changes of *cir *genes during intraerythrocytic development**. For expression profiling of the *cir *genes at three different time points in the life cycle, 30 μl tail vein blood of female NMRI mice infected with 100 pRBCs were collected 3 h (early trophozoites), 10 h (late trophozoites) and 17 h (mature trophozoites and early schizonts) after beginning of the light cycle on day 13 p.i. (parasitaemia about 30%). Amplification was performed using the subfamily-specific primers for both subfamilies and 150 ng of purified RT-PCR products (approx. 600 bp) were digested with *Alu*I and 30 ng were then analysed using the DNA 1000 kit for the Agilent 2100 bioanalyzer. The RFLP profiles of subfamily 1 are shown for four mice in (A). The restriction digests of subfamily 2 are shown in the upper panel of (B). Only a few restriction fragments were detected in all samples, therefore RT-PCR products were also restricted with *Xap*I (B, lower panel). DNA 1000 bp ladder was used and in each lane an upper (1000 bp, purple) and a lower (25 bp, green) marker were indicated.

For cir subfamily 1, changes in the transcription profile could be detected in the course of a life cycle in all four mice (Figure 9A). However, these changes were much smaller than the dramatic transcriptional changes in the course of an infection which were observed for two out of four mice in Figure 8A. Only minimal changes in the fragment pattern were also observed for cir subfamily 2. Because of the very simple and uninformative restriction fragment pattern for cir subfamily 2 cDNAs in all mice, an additional restriction digestion with *Xap*I was again performed (Figure 9B) likewise indicating only minimal differences in the transcription pattern of cir subfamily 2 during the intraerythrocytic life cycle of *P. chabaudi*.

## Discussion

In order to improve understanding of host/parasite interactions correlating antigenic variation and accumulation of parasite localization in different host tissues, expression of the *P. chabaudi cir *multigene family was analysed *in vivo *at the transcriptional level. Despite diverse *in vitro *or *in vivo *investigations regarding antigenic variation in *Plasmodium *species, the complete molecular mechanism of antigenic switching in the parasites is, hitherto, far away from being fully understood. The present study, therefore, examined *P. chabaudi *infections in immunocompetent mice to get further insight in the complex phenomenon of antigenic variation and sequestration.

In an initial phylogenetic analysis of the annotated repertoire of putative CIR proteins, two subfamilies and an unassigned group of very long CIRs could be identified. While the subfamily 1 and 2 exhibit the predicted primary structure of common PIR proteins, the unassigned CIR proteins show more divergence often due to large insertions within the conserved CIR-BIR-YIR domain.

It has already previously been proposed that different subsets within the *pir *superfamily exhibit different functions. In *P. yoelii *and *P. berghei*, for example, a stage specific role was assumed since not all *pir *genes are transcribed equally when different life cycle stages were compared [24,55]. Indeed, different roles for *P. chabaudi *cir subfamily 1 (with changes in expression pattern) and subfamily 2 (with minor or no changes in expression pattern) genes are likely. The large insertions and additional transmembrane domains found within the group of unassigned CIR proteins might also be interpreted as hints for distinct functions of these proteins. Since none of them has been examined in any functional or even localization study, there is still no clue for the role these proteins might play in the *Plasmodium *life cycle or in the pathogens immune evasion strategy. For the VIR proteins in *P. vivax *diverse protein domain and structure predictions have been suggested to indicate different functions for different subsets of these proteins as for example those with PEXEL motif or those with additional transmembrane domains [56]. However, the presence or absence of the PEXEL motifs might simply result in usage of alternative transport signals or pathways to the host cell membrane [57] and currently no functional role has been suggested for any of the PIR proteins with more than one transmembrane domain. Since bioinformatic analysis of the CIR proteins revealed not a single strong PEXEL motif, it can be assumed that PEXEL motifs are not an important feature in the function of CIR proteins and that at least the majority of PIR proteins is able to be transported to the pRBC surface without an obvious PEXEL translocation signal.

A common ancestry of the gene families forming the *pir *superfamily has been postulated due to e.g. conserved sequence motifs as well as structural predictions within the PIR sequences [20]. By phylogenetic comparison of CIR and YIR proteins as performed here, this hypothesis can be further strengthened. The phylogenetic relationship of the CIR proteins with the widely studied YIRs in *P. yoelii *shows that the YIR protein sequences only share high similarity to CIR proteins of subfamily 2 and neither to subfamily 1 nor the group of unassigned CIR proteins. Comparison to the YIR antigens of the close relative *P. yoelii *suggests that most of the CIR sequences in the *P. chabaudi *genome have evolved by diversification after separation of *P. yoelii *and *P. chabaudi *from a common ancestor. Such a dispersing evolution of distinctly evolved subfamilies has also been demonstrated by phylogenetic analysis of PIR proteins encoded in *P. yoelii *and *P. berghei *genomes [52] and it is likely that the high variability of antigens in the individual malaria species distinctly evolved probably in response of the host immune pressure.

In this study a non-clonal *P. chabaudi *line very similar to the clonal *P. chabaudi *AS strain was used. The deduced protein sequences of the amplified cir transcripts of these parasites, however, were quite similar but not identical to the annotated putative CIR predicted from the genome sequence of the clonal *P. chabaudi *AS strain supposing a high variability of antigens even between closely related strains within the same species.

Cloning and sequencing of a first subset of *cir *genes demonstrated that a broad range of subfamily 1 and subfamily 2 *cir *genes is transcribed during a *P. chabaudi *infection in immunocompetent mice infected with a starter population of a minimal size. These findings are consistent with those described previously for other *pir *multigene families, e.g. the *vir *genes in *P. vivax*, the *yir *genes in *P. yoelii *and the *bir *genes in *P. berghei *[25,26,55]. In contrast to the mutually exclusive expression of only one *var *gene found in *P. falciparum *parasites cultured *in vitro *[58], it has been shown that in *P. vivax *and in several rodents *Plasmodium *species many different *pir *genes were transcribed *in vivo *within a parasite population in an individual host. Interestingly, examinations of natural *P. falciparum *infected human samples has shown that - in contrast to the limited transcription pattern in cultured *P. falciparum *parasites - many transcripts of *var *and *stevor *genes are also transcribed simultaneously *in vivo *[59,60]. Such contradictory *in vivo *and *in vitro *findings make clear that *in vivo *models such as *P. chabaudi *or other rodent malaria parasites are essential for complex investigations of antigenic variation.

In accordance with previous studies of the *yir *genes of *P. yoelii *[24], switching of *cir *gene expression could be detected around peak parasitaemia in the course of infection suggesting that antigenic variation might be modulated by selective forces exercised by the host immune system. However, these transcriptional changes were not observed in all infected mice and, moreover, were not detectable to the same extent for all cir subfamilies. Most marked differences in mRNA expression patterns could be observed for cir subfamily 1 whereas no or obviously less transcriptional switching was detected for cir subfamily 2 during the infection.

Completely different cir gene expression patterns of the progenies derived from 100 pRBCs starter populations originating from the same parental population strongly suggest that a large parasite population can be extremely heterogeneous. In micromanipulated *P. yoelii *pRBCs, it has been shown that only one to three different *yir *transcripts were transcribed within an individual cell but many different transcripts were detected within a whole parasite population [25]. In addition, rapid switching in the transcribed repertoires of *yir *genes between different clonal host parasites populations and parasite developmental stages has been described. Although no infections with single pRBCs were performed in the present study, the initial diversity of parasites was apparently kept at a minimum using the minimal infectious dose resulting in patent infections by intraperitoneal infection as revealed by the substantial differences in RT-PCR RFLP patterns between mice infected with 100 pRBCs. Therefore, the number of transcribed *cir *genes per pRBC is presumably also much lower than that found to be transcribed in a large population.

Previous studies have analysed switching between different *yir *genes in *P. yoelii *infected mice both in immunocompetent [24] and highly immunodeficient [25] mice. Efficient and frequent switching of expressed *yir *genes could be observed even in the absence of any selecting force of an adaptive immune system [25]. The fact that *cir *gene subfamily 1 and subfamily 2 expression patterns apparently do not necessarily change in the course of a *P. chabaudi *infection in immunocompetent mice even within three weeks suggests lower on-off switching frequencies at least for these groups of *cir *genes. This is particularly surprising as adaptive immune responses are well known to effectively select for parasites expressing new variant antigens in other protozoan parasites [61-63].

Changes in the expression patterns of variant antigens appear to occur more frequently in cir subfamily 1 than in subfamily 2. No data are currently available for switching frequencies within the highly heterogeneous group of unassigned *cir *genes. Fonager *et al *[52] already speculated that within the *yir *gene family those genes belonging to highly heterogeneous groups would be more important for antigenic variation than members of more conventional subfamilies. The phylogenetic analysis including members of all previously defined *yir *groups [52] shows that even the most divergent YIR proteins cluster together with the CIR subfamily 2. This suggests that subfamily 2 is ancient among the CIR proteins while subfamily 1 and the unassigned CIR proteins have probably evolved from ancient genes by diversification. The higher apparent switching frequency observed for cir subfamily 1 than subfamily 2 genes is a first experimental hint corroborating the hypothesis that those *pir *family members that evolved relatively recently might play a more prominent role for antigenic variation than those showing ancient properties [52]. Future work in both *P. yoelii *and *P. chabaudi *should, therefore, no longer neglect these unusual *pir *members from the analyses but should pay special attention to them.

Analysis of changes in *cir *gene expression patterns in the course of one asexual round of multiplication in 24 h revealed only minor changes in gene expression for subfamily 1 and virtually no changes for subfamily 2. In particular, no reduction in the complexity of the *cir *genes in late parasite stages was observed in comparison to early rings as has been described for *var *genes [58]. Since Cunningham *et al *[25] found evidence that in *P. yoelii *even schizonts transcribe at least up to three different *yir *genes, a mutually exclusive expression of *pir *genes in late individual parasites or parasite populations appears to be highly unlikely. The same was also shown for *vir *genes in *P. vivax *with more than one antigen expressed in a single parasite and different expression patterns between parasites [26]. Transcriptomic analysis of *P. vivax *intraerythrocytic developmental cycle stages also revealed that many *vir *genes are expressed early during the ring stage and turned off later while others are expressed predominantly in schizonts [64]. These authors could not find any linkage between the position of the *vir *gene within the phylogenetic tree and its predominant expression time. In contrast to these results, only minor changes during the intraerythrocytic cycle were found in the present study. Possible explanations for this discrepancy include the fact that of course the RFLP analysis is less sensitive with regard to the detection of changes in expression of individual genes when compared to the microarray method used by Bozdech *et al *[64]. In addition, only for about 60% of the *vir *genes consistent temporal expression patterns could be observed for three different *P. vivax *isolates. If the highly expressed genes show no temporal expression pattern, such a pattern would clearly not be detectable with the RFLP method used here since it is not able to detect minor cir transcripts at all. Finally, the different experimental designs with only 100 pRBCs as founders in the *cir *gene study and non-selected parasites from naturally infected patients for the *P. vivax *transcriptome study might well contribute to the observed differences.

Remarkable differences in the mRNA expression patterns between pRBCs in blood, liver, spleen, kidney, lung and brain could be observed suggesting a host-tissue specific expression of *cir *genes. It is believed that the PIR proteins, like the Pfemp1 protein family in *P. falciparum*, are possibly involved in adhesion to host receptors thus mediating accumulation and sequestration in different host tissues. For the BIR or YIR proteins, for example, an expression of these molecules on the surface of pRBCs has already been demonstrated [24,27] but neither direct nor indirect evidence for a correlation of an adhesion of PIR proteins to host endothelial cells has yet been found. Accumulation of pRBCs in different tissues expressing different *cir *genes, however, can be considered to be a first experimental hint that CIR and maybe also other PIR proteins might indeed be involved in adhesion and sequestration. Whether rapid and dramatic changes in tissue distribution of *P. chabaudi *parasites at peak parasitaemia, i.e. exclusion from the splenic red pulp [65], has effects on *cir *gene expression patterns would also be interesting to analyse in the future. Since *P. chabaudi *is the only frequently used experimental malaria model with synchronous development and a strong sequestration phenotype, these results suggest that further studies of CIR proteins will provide important new insights into the interaction of non-*P. falciparum *malaria pRBC with host epithelia.

## Conclusions

The present study has demonstrated for the first time that there are differences in the tissue-specific expression of some *cir *genes. These results suggest a possible correlation between the expression of CIR antigens and accumulation of parasites in inner organs of the host. The high agreement in results obtained for transcriptional switching and antigenic variation of the *cir *genes with that for other members of the *pir *superfamily indicate very well that the *cir *genes are promising candidates for further functional studies. For example parasites expressing certain CIRs constitutively as transgenes might improve understanding of the functional role of the *pir *superfamily in *Plasmodium *infections.

## Competing interests

The authors declare that they have no competing interests.

## Authors' contributions

JK designed the study, planned and supervised all experiments. PE performed the experiments. JK and PE did bioinformatic and statistical analyses and wrote the manuscript. All authors read and approved the final version.

## Supplementary Material

Additional file 1 **All annotated putative CIR proteins from PlasmoDB**. This Excel file contains all 199 putative CIR proteins annotated in the PlasmoDB database (September 2011) including Gene ID numbers and several sequence details such as sequence sizes and protein motifs. The 13 partial CIR proteins excluded of the phylogenetic analysis are shaded in grey. For three proteins containing more than one CIR-BIR-YIR domain, only the complete domain was used. This is indicated by giving the position of this artificial truncation. un, unassigned; N/A, not available. Click here for file

Additional file 2 **Full-length *cir *genes cloned from the non-clonal *P. chabaudi *line**. This Exel file contains the GenBank Accession numbers as well as the primer pairs for amplification of 6 *cir *full-length sequences of the non-clonal *P. chabaudi *line. The amplification of these sequences was performed with the AccuPrime™ *Taq *DNA Polymerase (Invitrogen) with an initial denaturation for 30 s at 94°C, 40 cycles of 10 s at 94°C, 1 min at 45°C and 2 min at 72°C followed by a final extension for 10 min at 72°C. The PCR products of cDNA and genomic DNA were cloned, sequenced and aligned for verification of complete exon-intron structure of the *cir *genes. Click here for file

Additional file 3 **Phylogenetic tree of 186 annotated CIR and selected YIR proteins in Newick format**. This text file contains the phylogenetic maximum likelihood tree of the 186 annotated CIR and selected YIR proteins in Newick format including Gene ID numbers and maximum likelihood ratios as statistical support at the nodes. Click here for file

Additional file 4 **Splice variant of *cir *gene found by RT-PCR and genomic PCR**. The PDF file shows the alignment of two *cir *transcripts amplified by RT-PCR with their corresponding genomic DNA sequence. The first *cir *transcript shows the common *cir *gene structure with three exons. The second *cir *transcript, in contrast, represents a splice variant in which the usual start codon is eliminated resulting in an NH_2_-terminally truncated protein. Start codons and stop codons are highlighted in green and red, respectively. The coding exons are colour shaded in blue and the primer sequences are highlighted in yellow. Click here for file

Additional file 5 **The amplified repertoire of cir cDNAs of the cloning study**. In this Excel file all amplified cir cDNAs of the cloning study are given with their GenBank^® ^Accession numbers. The cir cDNAs are sorted according to their subgroups. The origin of sequences (host tissues) is highlighted by colours: Blood (red), liver (purple), spleen (blue), kidney (yellow) and brain (cyan). Click here for file

Additional file 6 **Phylogenetic relationship of protein sequences deduced from the cloning study with the PlasmoDB CIR domains**. This PDF file shows the phylogenetic maximum likelihood tree of the 186 putative conserved domains of CIRs and the 190 deduced CIR protein sequences of the cloning and sequencing study. The cir subfamily 1 and subfamily 2 are highlighted in purple and cyan, respectively. The deduced CIR protein sequences of the cloning and sequencing study are highlighted with dots in the subfamily-specific colour. Click here for file

Additional file 7 **Newick format of the phylogenetic tree of cir subfamily 1**. This text file contains the phylogenetic maximum likelihood tree of the deduced proteins from 216 cDNAs of cir subfamily 1 in Newick format. The individual names for each sequence are indicated. Click here for file

Additional file 8 **Newick format of the phylogenetic tree of cir subfamily 2**. This text file contains the phylogenetic maximum likelihood tree of the deduced proteins from 216 cDNAs of cir subfamily 2 in Newick format. The individual names for each sequence are indicated. Click here for file

Additional file 9 ***In silico *restriction of the amplified cir cDNAs of the cloning study**. The fragment sizes of the restricted cDNAs sequences of the cloning study, *in silico *digested with *Alu*I and *Xap*I, were shown in this Excel file. In the first table sheet the restriction fragments of the cir cDNAs of subfamily 1 were listed, in the second table sheet of subfamily 2, respectively. The tissue origins of sequences are highlighted by colours: Blood (red), liver (purple), spleen (blue), kidney (yellow) and brain (cyan). The cir cDNA sequences are sorted according the subgroups. Click here for file
