# Supplementary material for: Characterization and tissue-specific expression patterns of the Plasmodium chabaudi cir multigene family
Source: Malar J. 2011 Sep 19;10:272. doi: 10.1186/1475-2875-10-272 (PMC3189184; doi:10.1186/1475-2875-10-272)
Supplement: Additional file 4 — Splice variant of cir gene found by RT-PCR and genomic PCR. The PDF file shows the alignment of two cir transcripts amplified by RT-PCR with their corresponding genomic DNA sequence. The first cir transcript shows the common cir gene structure with three exons. The second cir transcript, in contrast, represents a splice variant in which the usual start codon is eliminated resulting in an NH2-terminally truncated protein. Start codons and stop codons are highlighted in green and red, respectively. The coding exons are colour shaded in blue and the primer sequences are highlighted in yellow. [file 1475-2875-10-272-S4.PDF]

Alternative splice variant of *cir* genes detected in RT-PCR

```
gDNA      1 gacataaagaaatttaatattcccttttaatcacatttattattattttacattaatccaattgttataaacagtaagtatatgcttaatcatatatatt
cDNA1     1 gacataaagaaatttaatattcccttttaatcacatttattattattttacattaatccaattgttataaacagtaagtatatgcttaatcatatatatt
cDNA2     1 gacataaagaaatttaatattcccttttaatcacatttattattattttacattaatccaattgttataaaca---------------------------

gDNA    101 tacaagtaacacatatttatttaggcgtaatatttataattaaatgccattataatgtctgaggaattggtatataaatttgttaaatataatatgtctt
cDNA1   101 tacaagtaacacatatttatttaggcgtaatatttataattaaatgccattataatgtctgaggaattg-------------------------------
cDNA2       ----------------------------------------------------------------------------------------------------

gDNA    201 catatgtgattaatatgctgaatcgcctataaattatattaattttcattttaataacatttatattaatacattcttttctttaattcgtaaatatatt
cDNA1       ----------------------------------------------------------------------------------------------------
cDNA2       ----------------------------------------------------------------------------------------------------

gDNA    301 cgtagtgtggagcaattaaatttgttgatgaaaatgttgtcttcgataaaaatactcaaaactatacgtttaaagatgagatattcaaagcttttttgttc
cDNA1   169 -----tgtggagcaattaaatttgttgatgaaaatgttgtcttcgataaaaatactcaaaactatacgtttaaagatgagatattcaaagcttttttgttc
cDNA2    74 -----tgtggagcaattaaatttgttgatgaaaatgttgtcttcgataaaaatactcaaaactatacgtttaaagatgagatattcaaagcttttttgttc

gDNA    401 tctcagtggaaaggatggaaaaggaaaatgtgatactaatagggaatttctcaactctggtattatgggattgctagaatatgttaagagtattgatgag
cDNA1   265 tctcagtggaaaggatggaaaaggaaaatgtgatactaatagggaatttctcaactctggtattatgggattgctagaatatgttaagagtattgatgag
cDNA2   169 tctcagtggaaaggatggaaaaggaaaatgtgatactaatagggaatttctcaactctggtattatgggattgctagaatatgttaagagtattgatgag

gDNA    501 gaagagttagatggtgataaacttgctcaatacgctattttatggtttaattataaaattagccaagatcagaatatagagattataagaggtactatgt
cDNA1   365 gaagagttagatggtgataaacttgctcaatacgctattttatggtttaattataaaattagccaagatcagaatatagagattataagaggtactatgt
cDNA2   269 gaagagttagatggtgataaacttgctcaatacgctattttatggtttaattataaaattagccaagatcagaatatagagattataagaggtactatgt

gDNA    601 atgaaatacttaaacaaaatgattggtttggagaacatagtgaatccatagaaaaaataaaagatatgatgggaattcattctgaatatttgaagagact
cDNA1   465 atgaaatacttaaacaaaatgattggtttggagaacatagtgaatccatagaaaaaataaaagatatgatgggaattcattctgaatatttgaagagact
cDNA2   369 atgaaatacttaaacaaaatgattggtttggagaacatagtgaatccatagaaaaaataaaagatatgatgggaattcattctgaatatttgaagagact

gDNA    701 ttatagattacttaaaggaatatgtgaaacaattaatacatgtaatggctcttcagacaccaataaatgcatagaaagtgctaaaaaatgtgttgattcg
cDNA1   565 ttatagattacttaaaggaatatgtgaaacaattaatacatgtaatggctcttcagacaccaataaatgcatagaaagtgctaaaaaatgtgttgattcg
cDNA2   469 ttatagattacttaaaggaatatgtgaaacaattaatacatgtaatggctcttcagacaccaataaatgcatagaaagtgctaaaaaatgtgttgattcg

gDNA    801 tatcattcatgtattattcaatttccctggaaagagatttgtaatccatattgtcgtgtgttgtcaaatttaaaaaaagattatgataaatttagagaag
cDNA1   665 tatcattcatgtattattcaatttccctggaaagagatttgtaatccatattgtcgtgtgttgtcaaatttaaaaaaagattatgataaatttagagaag
cDNA2   569 tatcattcatgtattattcaatttccctggaaagagatttgtaatccatattgtcgtgtgttgtcaaatttaaaaaaagattatgataaatttagagaag

gDNA    901 ctaacaataataaagatcaacttccaacttttacaatacccgaaggagcaaagagctgtgaagaaacatgtgcaaaaaaatggcaagaaccagaggctaa
cDNA1   765 ctaacaataataaagatcaacttccaacttttacaatacccgaaggagcaaggagctgtgaagaaacatgtgcaaaaaaatggcaagaaccagaggctaa
cDNA2   669 ctaacaataataaagatcaacttccaacttttacaatacccgaaggagcaaagagctgtgaagaaacatgtgcaaaaaaatggcaagaaccagaggctaa

gDNA   1001 ggaaccggcaattgatgtttcagaaataggtacacccccccggaattagtttaccaggcctaccagtaacccccacaagtataaacaatggaaataaacta
cDNA1   865 ggaaccggcaattgatgtttcagaaataggtacacccccccggaattagtttaccaggcctaccagtaacccccacaagtataaacaatggaaataaacta
cDNA2   769 ggaaccggcaattgatgtttcagaaataggtacacccccccggaattagtttaccaggcctaccagtaacccccacaagtataaacaatggaaataaacta

gDNA   1101 ccctacatcgcagtcccattaattttaatacccattattttaggaatttcatataaggtaagtattacaattcaaaaatataaatttaaaaaatatacat
cDNA1   965 ccctacatcgcagtcccattaattttaatacccattattttaggaatttcatataag-------------------------------------------
cDNA2   869 ccctacatcgcagtcccattaattttaatacccattattttaggaatttcatataag-------------------------------------------

gDNA   1201 tacaacatatttttgagtgcataaaaagacaaataattatatatttttatttttatgttagtatttaacacccgtatggcgaaaaaagtcgaaaagaaa
cDNA1  1020 ------------------------------------------------------------tatttaacacccgtatggcgaaaaaagtcgaaaagaaa
cDNA2   924 ------------------------------------------------------------tatttaacacccgtatggcgaaaaaagtcgaaaagaaa

gDNA   1301 agccatgaaaaagattataaatttgagtgatcaaaagagagcctaaaatggcgttacaaatgcattcatcgaaaagagccaattggaataattataaatt
cDNA1  1060 agccatgaaaaagattataaatttgagtgatcaaaagagagcctaaaatggcgttacaaatgcattcatcgaaaagagccaattggaataattataaatt
cDNA2   964 agccatgaaaaagattataaatttgagtgatcaaaagagagcctaaaatggcgttacaaatgcattcatcgaaaagagccaattggaataattataaatt

gDNA   1401 gggttgatgaaaaatgggattattaaatatatacaaaatatggaggataattctataccagttatta
cDNA1  1160 gggttgatgaaaaatgggattattaaatatatacaaaatatggaggataattctataccagttatta
cDNA2  1064 gggttgatgaaaaatgggattattaaatatatacaaaatatggaggataattctataccagttatta
```
