# Supplementary figures and images for: Characterization and tissue-specific expression patterns of the Plasmodium chabaudi cir multigene family
Source: Malar J. 2011 Sep 19;10:272. doi: 10.1186/1475-2875-10-272 (PMC3189184; doi:10.1186/1475-2875-10-272)

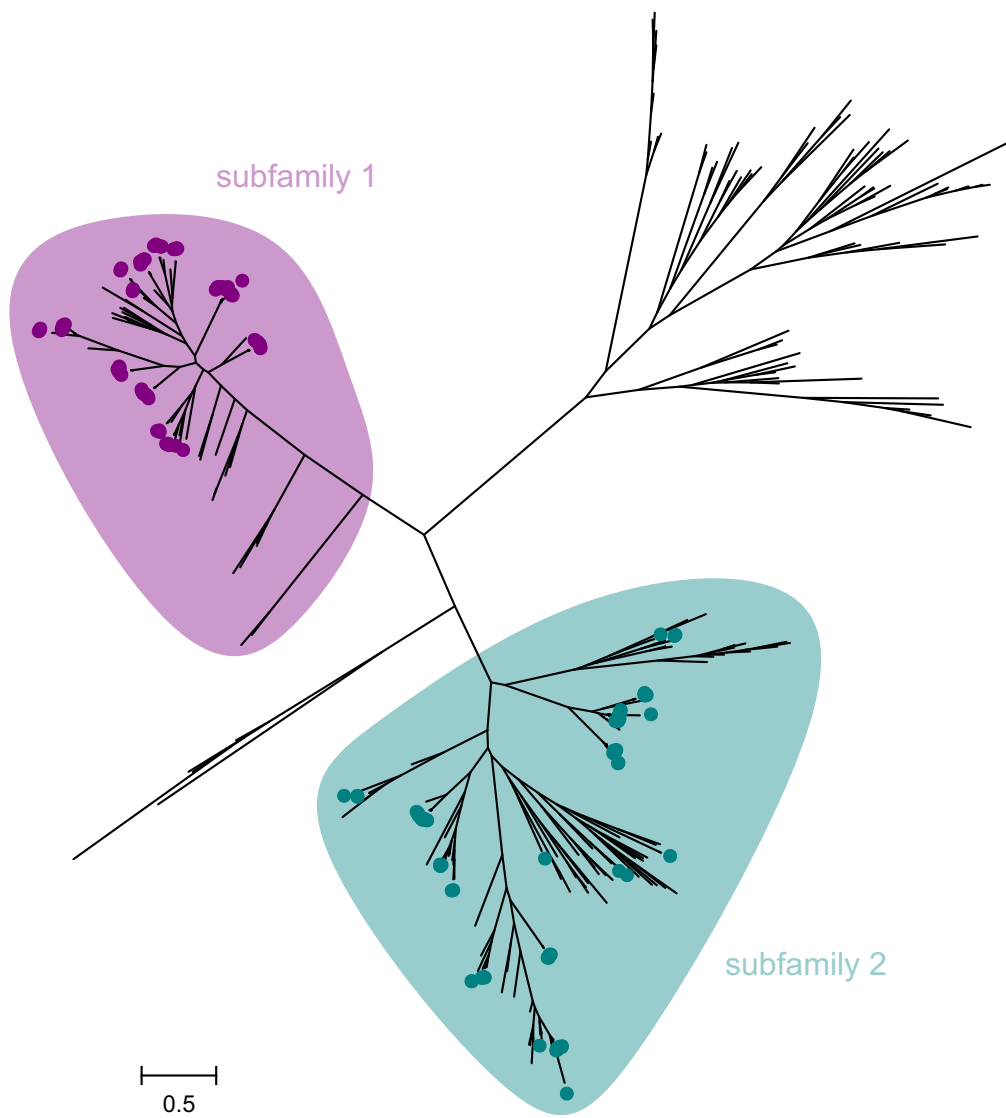

Supplement: Additional file 6 — Phylogenetic relationship of protein sequences deduced from the cloning study with the PlasmoDB CIR domains. This PDF file shows the phylogenetic maximum likelihood tree of the 186 putative conserved domains of CIRs and the 190 deduced CIR protein sequences of the cloning and sequencing study. The cir subfamily 1 and subfamily 2 are highlighted in purple and cyan, respectively. The deduced CIR protein sequences of the cloning and sequencing study are highlighted with dots in the subfamily-specific colour. [file 1475-2875-10-272-S6.PDF]
